# Identifying genes that contribute most to good classification in microarrays

**DOI:** 10.1186/1471-2105-7-407

**Published:** 2006-09-07

**Authors:** Stuart G Baker, Barnett S Kramer

**Affiliations:** 1 Biometry Research Group, Division of Cancer Prevention, National Cancer Institute, Bethesda, MD 20892-7354, USA; 2 Office of Disease Prevention, National Institutes of Health, Bethesda, MD 20892, USA

## Abstract

**Background:**

The goal of most microarray studies is either the identification of genes that are most differentially expressed or the creation of a good classification rule. The disadvantage of the former is that it ignores the importance of gene interactions; the disadvantage of the latter is that it often does not provide a sufficient focus for further investigation because many genes may be included by chance. Our strategy is to search for classification rules that perform well with few genes and, if they are found, identify genes that occur relatively frequently under multiple random validation (random splits into training and test samples).

**Results:**

We analyzed data from four published studies related to cancer. For classification we used a filter with a nearest centroid rule that is easy to implement and has been previously shown to perform well. To comprehensively measure classification performance we used receiver operating characteristic curves. In the three data sets with good classification performance, the classification rules for 5 genes were only slightly worse than for 20 or 50 genes and somewhat better than for 1 gene. In two of these data sets, one or two genes had relatively high frequencies not noticeable with rules involving 20 or 50 genes: desmin for classifying colon cancer versus normal tissue; and zyxin and secretory granule proteoglycan genes for classifying two types of leukemia.

**Conclusion:**

Using multiple random validation, investigators should look for classification rules that perform well with few genes and select, for further study, genes with relatively high frequencies of occurrence in these classification rules.

## Background

An important goal of microarray studies with two classes is to identify genes, for further investigation, that "explain" much of the class differences. One common approach is to look separately at genes that exhibit high differential expression. The disadvantage of this approach is that it ignores the role of gene combinations in leading to good classification. Another approach is to investigate classification rules for combinations of genes. While this approach accounts for gene interactions, too often these rules involve so many genes that it is difficult to determine those genes that are not included simply by chance. Our strategy is to look for classification rules that perform well with few genes, so as to have a stronger "signal" to detect genes that contribute most to good classification.

There are various approaches to developing classification rules for microarrays and identifying genes for further investigation. Almost all approaches involve a split of the data into training and test samples. In the training sample a classification rule is developed, and in the test sample its performance is determined. Two common measures of performance are cross-validation [1] and multiple random validation [2]. In k-fold cross-validation, the data are divided into k subsets. On each iteration of the cross-validation a different collection of the k-1 subsets serve as the training sample and the remaining subset serves as the test sample. The performance of the classification rule is the average performance in the k test samples [1]. In multiple random validation, the data are randomly split into training and test samples many times. Unlike cross-validation, the goal is not to average performance over test samples but to investigate the variability of performance over test samples and the frequencies of genes selected on random splits [2].

There are also various approaches for formulating a classification rule in the training sample. One common approach, called a filter, selects genes with the best individual performances prior to formulation of the classification rule. Another common approach, called a wrapper, splits the training sample into a training-training sample and a training-test sample and uses cross-validation *within *the training sample to both select genes and formulate a classification rule [3]. Unlike a filter, a wrapper can identify genes that perform poorly individually but well together, but the price is likely increased variability in the measure of performance due to a small training-test sample (although to our knowledge this has not been investigated). Importantly, both approaches for gene selection involve a threshold for the number of genes to include in a classification rule. Without a threshold it is possible to obtain excellent classification in the training sample (for example categorizing gene expression levels and ranking cross-classified cells by the ratio of true to false positives [4]) that would likely do poorly in the test sample because the classification rule has been overly tailored to the training sample. The threshold is either specified in advance or determined by a performance criterion and typically yields a moderate to large number of genes [2,5-8]

The identification of a moderate number of genes is theoretically desirable because the genes likely form a network. However we are concerned that, despite the use of refined approaches, it is more likely that with classification rules involving a moderate number of genes rather than a few genes, that some genes will be detected by chance Therefore our goal was to identify classification rules that perform well with the fewest genes, and so may be more "robust" than rules with more genes. Importantly with fewer genes in the classification rule, it is easier to identify genes with relatively high frequencies in multiple random validation. Such genes are most likely to represent a true "signal." The identification of a few genes that contribute to good classification and are not likely detected by chance allows investigators to better focus further research efforts, perhaps leading to easier clinical application, simpler dissemination of results, and possibly improved scientific insights.

Although our general strategy could be applied to any of a variety of microarray classification techniques, we chose a simple approach. For classification we used a filter and the nearest centroid rule, which is easy to implement and has been previously shown to perform well [9]. To measure performance in a comprehensive manner that captures errors in assignments to both classes, we used receiver operating characteristic (ROC) curves and the estimated area under the ROC curve (AUC), which ranges from 1/2 for chance classification to 1 for perfect classification [10]. To characterize chance variability we used the previously mentioned multiple random validation procedure that involves repeated random splits of training and test samples [2].

We analyzed data from four published microarray studies involving colon cancer [11], leukemia [12], medulloblastoma [13], and breast cancer [14]. See Table 1. Although multiple random validation strategy had been used with the colon cancer, medulloblastoma, and breast cancer data sets, these analyses did not involve a comparison of performances for few (less than 20) versus many genes.

**Table 1:** Description of studies

Data set	Number of genes	Number per class
Colon cancer [11]	2000	22 normal 40 tumor
Leukemia [12]	7219	47 acute lymphoblastic leukemia 45 acute myeloid leukemia
Medulloblastoma [13]	7129	39 survivors over two years 21 deaths over two years
Breast cancer [14]	7129	25 estrogen receptor positive 24 estrogen receptor negative

## Results

**Figure 1:**
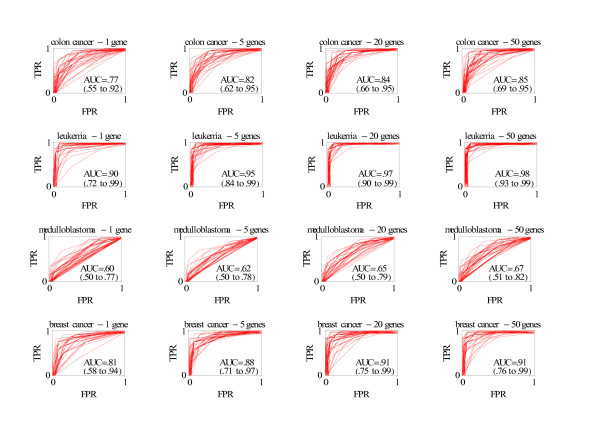
Smoothed ROC curves in test sample derived from multiple splitting of training and test samples. Graphs depict 40 randomly selected ROC curves out of 1000 splits. AUC is the mean area under the ROC curve from 1000 splits (95% confidence interval). FPR is false positive rate (one minus specificity) and TPR is true positive rate (sensitivity).

The classification performance generally improved as the number of genes in the classification rule increased from 1 to 5 to 20 to 50 with small decreases in improvement likely due to chance. See Figure 1 and Table 2. Because the classification for the medulloblastoma data was poor with an estimated AUC not significantly better than chance (Table 2), we only report in detail the results for the three other data sets. For these data sets, the performance of the classification rule was more similar between rules with 5, 20, and 50 genes than between rules with 1 and 5 genes, particularly when considering the lower bound of the estimated AUC. Results when the training sample split was one half the data are given below. For the colon cancer data set the estimated AUC (95% confidence interval) was .77 (.55 to .99), .82 (.62 to .95), .84 (.66 to .95), and .85 (.69 to .95) with 1, 5, 20, and 50 genes respectively; for leukemia data set it was .90 (.72 to 1), .95 (.84 to .99), .97 (.90 to .99), and .98 (.93 to 1) for 1, 5, 20, and 50 genes, respectively; and for the breast cancer data set it was .81 (.58 to .99), .88 (.71 to .97), .91 (.75 to .99), and .92 (.76 to .99) for 1, 5, 20, and 50 genes, respectively. Similar results were obtained with a four-fifths split into the training sample (Table 2).

**Table 2:** Estimates of area under ROC curve (AUC) and 95% confidence intervals

		Number of genes in classification rule			
Data set	Percent in training sample	1 gene	5 genes	20 genes	50 genes

Colon cancer	50%	.77 (.55 to .92)	.82 (.62 to .95)	.84 (.66 to .95)	.85 (.69 to .95)
	80%	.86 (.59 to 1)	.90 (.69 to 1)	.89 (.69 to 1)	.90 (.70 to 1)
Leukemia	50%	.90 (.72 to 1)	.95 (.84 to .99)	.97 (.90 to .99)	.98 (.93 to 1)
	80%	.95 (.76 to 1)	.97 (.84 to 1)	.99 (.91 to 1)	.99 (.93 to 1)
Medulloblastoma	50%	.60 (.50 to .77)	.62 (.50 to .78)	.65 (.50 to .79)	.67 (.51 to .82)
	80%	.65 (.50 to .88)	.69 (.50 to .88)	.75 (.50 to .94)	.78 (.50 to .97)
Breast Cancer	50%	.81 (.58 to .94)	.88 (.71 to .97)	.91 (.75 to .99)	.91 (.76 to .99)
	80%	.85 (.62 to 1)	.93 (.78 to 1)	.96 (.80 to 1)	.95 (.78 to 1)

For the three data sets in which the classification rule for 5 genes performed well, we investigated the classification rule for 5 genes to determine if any genes had relatively high frequencies of selection. Gene histograms are shown in Figure 1. For the breast cancer data set, no gene had a relatively high frequency. For the colon cancer data set, the human desmin gene had a relatively high frequency (57% versus the next four highest of 32%, 28%, 28% and 26% when training sample contained half the data and 97% versus the next highest of 52%, 50%, 49%, and 45% when the training sample contained four-fifths of the data). Human desmin is essential for maintaining the structural integrity of skeletal muscle [15]. Desmin has also been used in a study of colon cancer as a marker for fat storing cells [16], and, in a study of colon polyps from 10 cases, all were negative for desmin [17].

For the leukemia data the zyxin gene had a high relative frequency (82% versus the next four highest of 42%, 37%,30% and 20% when the training sample contained half the data and 100% versus the next four highest of 82% (which we also discuss), 44%, 34%, and 31% when the training sample contained four-fifths the data.) Zyxin plays a pivotal role in mitosis [18]. After completing our analysis, we found that other investigators of the same data set identified zyxin has the most significant feature for classification, discussed some possible biological links, and recommended further investigation of the role of zyxin in leukemia [19].

Secretory granule proteoglycan was the other gene with high relative frequency in the leukemia data set when the training sample contained four-fifths of the data. The peptide core of a secretory granule proteoglycan, serglycin, has been implicated in gene methylation of leukemia cells [20]. Methylation is an important process in the regulation of expression in many genes. Serglycin is also involved in the formation of granules in neutrophil granulocytes [21]. Granules are important for distinguishing the two classes of leukemias. Interestingly secretory granule proteoglycan was not listed among identified genes in other classifications of these data [8,19].

## Discussion

Other classification rules for the same data sets performed similarly to ours [2,7,12]; however precise comparison of misclassification rates is not meaningful due to differences in the validation procedures, particularly when there was no random splitting of training and test samples. In terms of methodology for gene selection and classification, there are several related approaches: a filter with multiple random validation of the entire sample [2], a wrapper with multiple random validation of the entire sample [7], and a wrapper with multiple random cross-validation *within *the training sample and no independent test sample [8] (so there is no clearly unbiased estimate of classification performance). See Table 3. Our approach can be viewed either as (i) a wrapper with multiple random validation (instead of cross-validation) and a training-test sample instead of a test sample or (ii) a filter with multiple random validation of the entire sample. These related approaches computed either gene frequencies or a relevancy measure [8] that can be viewed as a frequency when the weights equal 1. Our general strategy of finding classification rules with few genes that perform well and then identifying genes with relatively high frequencies under multiple random validation applies to both classification rules with filters and wrappers. Future research using a wrapper would be of great interest because of the potential of the wrapper to identify genes that have good classification when considered together but poor classification when considered separately.

**Table 3:** Comparison of related methods.

Authors	Training sample	Test sample	Random aspect	Results
Michiels et al, 2005 [2]	(1) Selected genes most correlated with prognosis, (2) Created nearest centroid classification rule.	Used	Test and training sample splits in entire data set.	(1) Misclassification rate for test samples (2) Frequencies of genes selected in training sample
Ma et al, 2006 [7]	(1) Split into training-training sample and training-test sample, (2) Using cross-validation, maximized the binormal area under ROC curve as a linear function of genes; then selected genes with non-zero coefficients.	Used	Training-training and training-test samples (i.e. the cross-validation and evaluation is repeated)	(1) Area under ROC curve for test samples, (2) Frequencies of genes selected in training sample.
Li et al, 2004 [8]	(1) Split into training-training sample and training-test sample, (2) Cross-validated classification tree to maximize fit.	Not used	Resampling for training-training samples and training test samples.	(1) Relevancy intensity, which equals frequencies of genes selected in training sample when weights equal 1.
Proposed method	(1) Selected genes with highest individual, classification performance (2) Created classification rule using nearest centroid and score function.	Used	Test and training samples splits in entire data set.	(1) ROC curve and area under ROC curve for test samples with emphasis on comparing many versus few genes, (2) Frequencies of genes selected in training sample.

The inclusion of additional genes in the classification rule can affect performance in one of two ways depending on whether or not the additional genes are predictive of outcome or not. If the additional genes are not predictive (being selected by chance), the performance of the rule in the test sample will likely worsen due to additional "noise". If the additional genes are predictive, then performance in the test sample will improve with more "signals." Improvement in performance is greatest if the additional predictive genes are independent and smaller if the additional predictive genes are correlated. In our microarray studies, which showed small gains in performance with additional genes, the genes were likely predictive and correlated.

Our goal is to identify genes with high relative frequencies of selection in the classification rule with few genes. It is important to consider only classification rules with few genes because with moderate numbers of genes in the classification rules, many genes invariably appear in nearly all the classification rule and hence there is a concern that many genes are included by chance. With few genes in the classification rule, one can sometimes find, as in two of our data sets, one or two genes with relatively high frequencies, which seems like a strong "signal" that these genes make a real contribution to classification and are hence worthy of further study. It is, however, possible that many highly associated genes could have similarly high frequencies of occurrence in classification rules with few genes and one should be aware of this potential scenario.

Throughout this study we have been "conservative" in our identification of genes for further study by trying to rule out, as much as possible, the role of chance in explaining good classification. Hence we used multiple random validation and focused on as few genes as possible. We also wanted our measure of performance to be as informative as possible by using ROC curves. The 95% confidence intervals we report are only approximate because they are based on repeated sampling from a finite population, namely 49 to 92 specimens. However for our purposes, approximate confidence intervals are acceptable, because the main focus is not precisely estimating the variability of classification performance but rather the identification of genes that make a strong contribution to good classification performance.

## Conclusion

A relevant quote attributed to the noted mathematician Henri Poincare is, "Science is built up of facts, as a house is with stones. But a collection of facts is no more a science than a heap of stones is a house." Often investigators report large numbers of genes that are either differentially expressed or constitute a classification rule, but which, due to the influence of chance, may be more of a "heap of stones" than part of a "house." To confidently identify the basic "building blocks" for classification of cancer outcomes, investigators should use multiple random validation to find classification rules that perform well with few genes and select genes with relatively high frequencies of occurrence in these classification rules.

## Methods

The data were repeatedly randomly split into training and test samples. We investigated a both 50-50 and a 80-20 split for the training and test samples. For the single gene filter applied to the training sample, genes were ranked by the absolute value of the difference in mean levels between classes divided by the estimated standard error. The highest ranking genes of a specified number (1, 5, 20, or 50), were used to create the classification rule. For the selected set of genes in the training sample, the centroid (a list of average values for each gene expression level across all specimens in the sample) was computed for each class (designated 0 or 1) [9]. Thus the classification rule was a list of genes and their centroids for each class. Let d_0i _(d_1i_) denote the distance from the i^th ^test sample specimen to the class 0 (1) centroid in the training sample where distance is the square root of the sum (over genes in the rule) of the squared differences between the gene level in test sample specimen and the training sample centroid. The score for the i^th ^test sample specimen was d_0i_/(d_0i_+d_1i_).

Performance was measured using an ROC curve computed by applying the classification rule developed in the training sample to the complementary test sample. The ROC curve plots the false positive rate (FPR), which equals one minus specificity, versus the true positive rate (TPR) which equals sensitivity. An ROC curve is more informative than total misclassification error (the sum of one minus TPR and FPR), which is a commonly used outcome measure for classification studies with microarray data. For each random split into training and test samples a new ROC curve was generated in the test sample.

The part of the ROC curve of interest depends on the purpose of the study. For early detection of cancer only a small part of the ROC curve is of interest, the portion with a very low FPR [22]. For these studies involving the classification of cancer patients, as in the data sets used here, the entire ROC curve is of interest as well as the area under the curve (AUC), which is the area between the ROC curve and horizontal axis. A very good classifier has a high true positive rate for a given false positive rate, so that the ROC curve occupies the upper left hand side of the graph with an AUC approaching the ideal of 1.0. A classifier with no predictive values beyond random chance follows the diagonal from false and true positive rates of 0 to false and true positive rates of 1 and has an AUC of 0.5. The AUC can also be interpreted as the true positive rate averaged uniformly over the range of false positives [10]

A preliminary ROC curve was obtained by dividing the score into cutpoints and computing sensitivity and specificity for each cutpoint. To obtain a final ROC curve, the preliminary ROC curve was smoothed by taking, as the next successive point from left to right, the midpoint between the two points with the steepest slope from the previous point. Our measure of performance, the area under the ROC curve (AUC), was computed as the sum of the trapezoids [23].

The random split of training and test samples was repeated 1000 times, creating a set of 1000 ROC curves and a list of 1000 AUC's. A random sample of 40 ROC curves was graphically displayed along with the mean AUC and the 95% confidence interval for the 1000 splits. The lower (upper) bound of the 95% confidence interval was the value of the AUC at the 2.5 (97.5) percentile of the distribution of the 1000 AUC values. When lines from ROC curves coincided with the axes, a small random error away from the axis was added so that the thickness would indicate multiple lines. Another outcome measure was the frequencies of genes selected in the training sample [2].

## Authors' contributions

SGB wrote the computer code and drafted the manuscript. BSK provided substantive comments and refined the manuscript. All authors read and approved the final manuscript.

**Figure 2:**
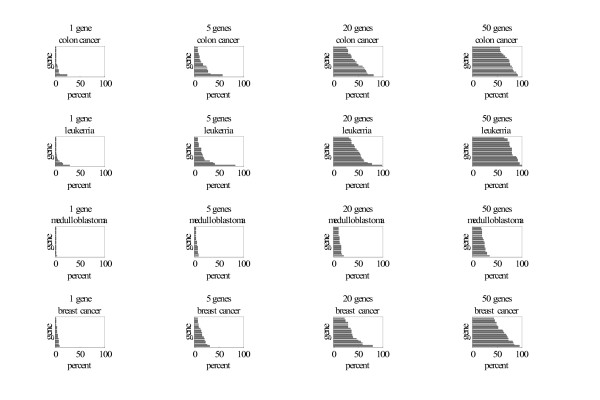
Histograms of the 20 genes selected most frequently in 1000 randomly selected training samples when forming classification rules involving 1, 5, 20, and 50 genes. The horizontal axis is the percent of all classification rules (with the indicated number of genes) for which the gene appears. Each horizontal bar represents a different gene.
